# In Vitro Evaluation of Olive Leaf (*Olea europaea* L.) Extract as a Functional Food Component in Combination with Chemotherapeutics in MCF-7 Breast Cancer Cells

**DOI:** 10.3390/ph18070965

**Published:** 2025-06-27

**Authors:** Eda Büker, Fadime Kiran, Seval Taliboglu, Dorina Casoni, Ayşe Ipekel

**Affiliations:** 1 Department of Basic Pharmaceutical Sciences, Faculty of Pharmacy, Gazi University, Ankara 06330, Türkiye; 2 Pharmabiotic Technologies Research Laboratory, Department of Biology, Faculty of Science, Ankara University, Ankara 06100, Türkiye; fkiran@science.ankara.edu.tr (F.K.); seval_taliboglu99@hotmail.com (S.T.); 3 Department of Chemistry, Faculty of Chemistry and Chemical Engineering, Babes-Bolyai University, Arany János, No. 11, RO-400028 Cluj-Napoca, Romania; 4 Research Centre for Advanced Chemical Analysis, Instrumentation and Chemometrics–Analytica, Babes-Bolyai University, 11 Arany Janos Str., RO-400028 Cluj-Napoca, Romania; 5 Medical Farm, Mestanli Koyu 45930, Türkiye; ayseipekel@hotmail.com

**Keywords:** breast cancer, chemotherapeutic drugs, functional food, *Olea europaea* L., synergistic effect, vitamin E, water-based natural extract

## Abstract

**Background**: Since breast cancer is a major cause of mortality, investigation of the synergistic effect of *Olea europaea* L. leaf extract in combination with some cancer medications is important for obtaining cost-effective and high-achieving treatments for breast cancer. This study aims to investigate the potential effects of *Olea europaea* L. extract in inhibiting breast cancer cell growth and enhancing the efficacy of chemotherapy agents against breast cancer under in vitro conditions. **Methods**: We conducted an analysis of some minerals and vitamins of three different viscosities (200 V, 300 V, and 400 V as a natural food product) of *Olea europaea* L. leaf water-based extract (OWE) derived from a natural cold maceration. We investigated the cytotoxic effects of *Olea europaea* L. extract with different viscosities (200–400 V) and various chemotherapy agents, either alone or in combination, in estrogen receptor-positive MCF-7 human breast carcinoma cells by MTT assay. *Olea europaea* L. extract treatment of cells resulted in growth inhibition in a dose- and time-dependent manner. **Results**: The 400 V OWE showed the highest calcium (301 ± 12 mg/100 g), potassium (1744 ± 33 mg/100 g), and vitamin E (0.36 ± 0.01 mg/100 g) amounts. Based on MTT results, combinations of 400V *Olea europaea* L. extract, which exhibited the strongest inhibitory effect with an IC_50_ value of 940 µg/mL, and anticancer drugs were next assessed for their synergistic efficacy towards cell growth inhibition. **Conclusions**: Combinations of the IC_50_ value of 400 V OWE with docetaxel, paclitaxel, and trastuzumab (1 µg/mL) treatment showed a strong synergistic effect in the growth inhibition of MCF-7 cells.

## 1. Introduction

Cancer stands as the foremost cause of global mortality. In 2022, the International Agency for Research on Cancer (IARC) estimated that there were 19.9 million cancer cases worldwide. Breast cancer comprises 11.5% of all types of cancer all over the world (International Agency for Research on Cancer). Cancer treatment has advanced, but full recovery often requires a comprehensive approach involving surgery, chemotherapy, radiotherapy, and immunotherapy [1]. Cancers linked to factors such as poor diet, physical inactivity, smoking, etc., including breast, lung, and colorectal cancers, are already increasing in economically transitioning nations [2,3].

Breast cancer is a significant global health concern, with over 1.6 million new cases and more than 5 hundred thousand deaths annually [4]. In the last few years, the FDA has approved more drugs for breast cancer indications than for any other type of cancer [1]. Docetaxel, doxorubicin, paclitaxel, and trastuzumab are some of the drugs specifically approved by the FDA for either a single or adjuvant treatment of breast cancer [5]. Docetaxel demonstrates antineoplastic (anticancer) activity against a wide variety of cancer cells, shows synergistic effects when used in combination with several other anticancer agents, and often exhibits greater cytotoxic activity against human breast cancer cell lines compared to paclitaxel [6]. Paclitaxel has the remarkable ability to trigger programmed cell death, known as apoptosis, in vulnerable cells. Preclinical studies have demonstrated its effectiveness against a wide range of tumors, such as ovarian and breast cancer [7,8]. Doxorubicin is an anthracycline that produces harmful radicals during metabolism that cause lipid peroxidation and can lead to RBC hemolytic lesions due to iron overload [9,10,11]. Trastuzumab, targeting HER-2, improves survival in metastatic disease when used with chemotherapy. It increases response rates and overall survival by 30% but may cause cardiac dysfunction, especially with anthracyclines [12].

Breast cancer stands as the second most prevalent cancer type among women globally. The diagnosis often necessitates an intensive chemotherapy regimen, which can significantly impact a patient’s mental health, physical condition, and overall quality of life (QOL). In the pursuit of curing cancer, aggressive multi-agent treatments can lead to considerable adverse effects, including nausea, vomiting, fatigue, hair loss, peripheral neuropathy, and cognitive impairment. Women undergoing chemotherapy for breast cancer may also experience diminished sexual function and premature menopause, alongside disruptions in central nervous system function. Doxorubicin, while effective in targeting rapidly dividing cancer cells, poses risks such as cognitive impairment. Similarly, docetaxel and paclitaxel are associated with infertility and peripheral neuropathy. The cardiotoxicity of doxorubicin arises from the formation of free radicals during its metabolism, leading to damage to cardiomyocytes. Palmar–plantar erythrodysesthesia (PPE), also known as acral erythema or hand–foot syndrome, is an adverse reaction linked to paclitaxel, occurring with an incidence rate of 1.5% to 3.2%. Other medications, including doxorubicin, cytarabine, and 5-fluorouracil, can also cause PPE. After chemotherapy, breast cancer patients often experience a significant drop in serum vitamin D levels, with levels between 20 ng/mL and 50 ng/mL considered adequate for healthy individuals, while levels below 12 ng/mL indicate a deficiency. A major complication of treatment is febrile neutropenia (FN), which can lead to serious morbidity and mortality. The development of FN may influence decisions regarding future chemotherapy, potentially compromising treatment effectiveness. The risk of FN is influenced by the intensity and duration of chemotherapy, as well as patient-specific factors such as age, serum albumin levels, and comorbid conditions [13].

Breast cancer incurs the highest treatment costs of any cancer in the United States, and these expenses are expected to increase. It is known that treatment costs, resource utilization, and patient outcomes differ based on the stage at diagnosis. Significantly, annual per capita inpatient expenditures for patients experiencing D/F care are over USD 5000 higher [14]. In the 24 months following diagnosis, the average costs permitted per patient are USD 71,909, USD 97,066, USD 159,442, and USD 182,655 for disease stages 0, I/II, III, and IV, respectively [15].

While new therapies for breast cancer are being developed to decrease the adverse effects and the cost of the treatments, there remains a need for enhanced treatment approaches. Plant-derived natural compounds are considered a new frontier in inducing immunogenic cell death (ICD) for cancer treatment [16]. A growing number of ICD inducers from natural products with promising anticancer potential have been identified in recent years, and an emerging focus on natural products is actually revealed in this area [17]. Moreover the therapeutic efficacy of combinations of some natural products with classical chemotherapy has been the subject of some clinical investigations in recent years [18,19]. Olive leaves as a functional food, abundant in the Mediterranean basin, are a rich source of beneficial phytochemicals and have been linked to health benefits in phytotherapy. Studies have shown that people following a Mediterranean diet have a lower incidence of certain types of cancer, including breast, skin, and colon cancer [20]. Olive leaves and their extracts have been utilized for their health-promoting properties and are marketed for immune system enhancement, antibiotic effects, and anti-aging benefits [21]. The key bioactive compounds found in olive leaf extracts, such as oleuropein and hydroxytyrosol, contribute to their potential health benefits [22]. Research has demonstrated that olive leaf extracts possess anticancer, antiproliferative, and antioxidant properties [23,24]. Previous studies have shown the effectiveness of olive leaf extract and its components in treating breast, skin, and bladder cancers in laboratory settings [25,26]. A recent in vivo study in male Sprague–Dawley rats revealed the significant antioxidant effects of olive leaf extract (OLE), reducing doxorubicin-induced cardiac, hepatic, and renal oxidative stress and injury [27].

This study aimed to determine whether varying viscosities of *Olea europaea* L. leaf water-based extract (OWE) could enhance the therapeutic efficacy and potential treatment outcomes for breast cancer, providing pre-information on innovative in vivo experiments for cancer therapy. In our comprehensive research investigation, we thoroughly analyzed three different viscosities of OWE (200 V, 300 V, and 400 V, as a natural food) in terms of minerals (calcium (Ca), ferritin (Fe), magnesium (Mg), manganese (Mn), phosphorus (P), potassium (K), selenium (Se), sodium (Na), and zinc (Zn)) and vitamins (A, B5, C, D2, E, and K1) and examined the cytotoxic effects of three different viscosities of OWE when administered independently and in conjunction with each of the following chemotherapy drugs, docetaxel, doxorubicin, paclitaxel, and trastuzumab, on the MCF-7 estrogen receptor-positive breast cancer cell line by MTT assay.

## 2. Results

In this study, the antiproliferative and cytotoxic effects of *Olea europaea* L. extract on breast cancer cells were evaluated and its potential synergistic effects with cancer drugs docetaxel, doxorubicin, paclitaxel, and trastuzumab was investigated. The minerals and vitamins that are important both alone and in combination with cancer treatment medications were determined in *Olea europaea* L. extracts with different viscosities.

### 2.1. Results of Minerals and Vitamins Analysis of OWE

In this study the mineral (Ca, Fe, Mg, Mn, P, K, Se, Na, and Zn) and vitamin (A, B_5_, C, D_2_, E, and K_1_) content for three different viscosities of OWE (200 V, 300 V, and 400 V) was determined. The obtained results presented in Table 1 show that potassium (1744 ± 33 mg/100 g), phosphorus (218 ± 7 mg/100 g), and calcium (301 ± 12 mg/100 g) were highest in OWE 400 V. Regarding vitamins A, B5, C, D2, E, and K1, the three different viscosities of OWE exhibited varying levels, detailed in the table. OWE 400 V has the highest vitamin E value of 0.36 ± 0.01 mg/100 g compared to OWE 200 V and OWE 300 V. OWE 300 V showed the highest quantity of vitamin C. According to these results, OWE 200 V has the highest ferritin value of 5.50 ± 0.50 mg/100 g.

### 2.2. Cytotoxic Effect of Olea europaea L. Extract and Drugs

**Figure 1:**
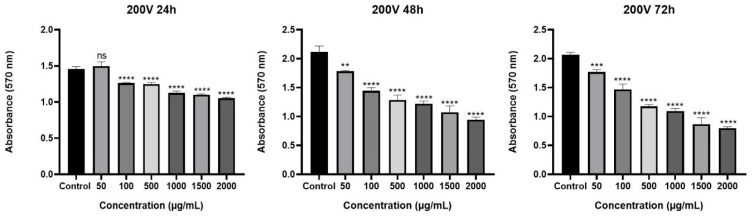

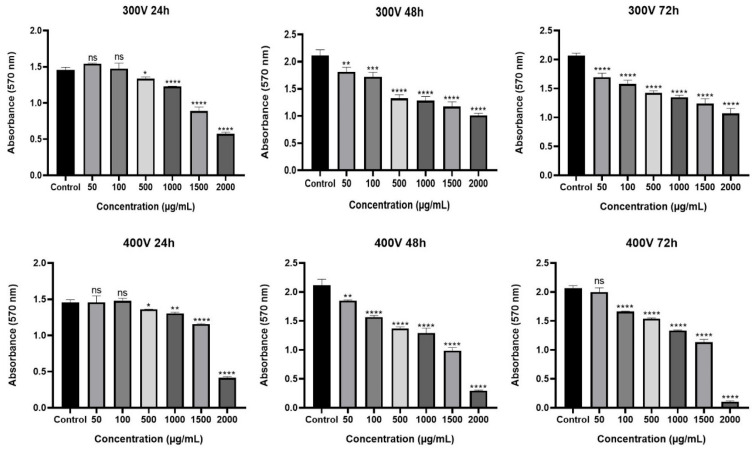
The impact of *Olea europaea* L. extracts of varying viscosities and concentrations on cell viability of MCF-7 after treatment periods of 24, 48, and 72 h (ns: nonsignificant, * *p* < 0.05, ** *p* < 0.01, *** *p* < 0.001, **** *p* < 0.0001).

**Figure 2:**
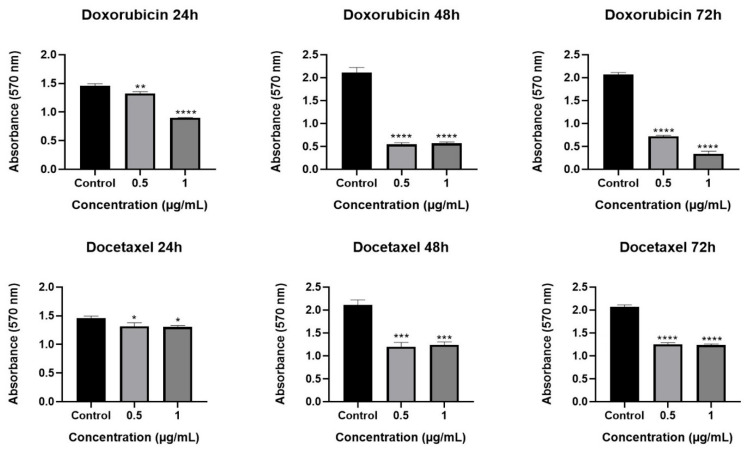

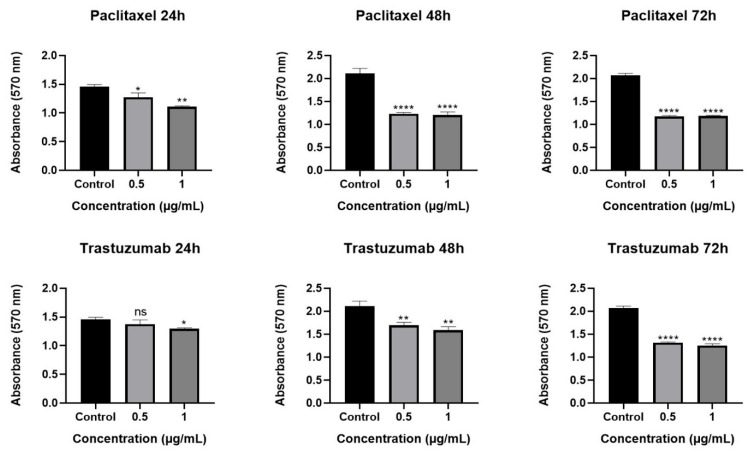
The effects of drugs on cell viability of MCF-7 following treatment periods of 24, 48, and 72 h (ns: nonsignificant, * *p* < 0.05, ** *p* < 0.01, *** *p* < 0.001, **** *p* < 0.0001).

The cytotoxic effect of *Olea europaea* L. extract was determined by MTT assay. Our results showed that *Olea europaea* L. extract decreased the viability of MCF-7 cells in a dose- and time-dependent manner after 24, 48, and 72 h of treatment (Figure 1). When the viscosity of the extracts was increased, a greater decrease in cell viability was observed. In particular, cells treated with the highest viscosity of *Olea europaea* L. extract (400 V) showed almost a 90% reduction (*p* < 0.0001) in cell viability with a concentration of 2.000 µg/mL and were selected for further analysis. The IC_50_ dose was found to be 940 µg/mL and used in the co-treatment strategies. When the results were considered in terms of the cytotoxic effect of the tested drugs, it was shown that a 1 µg/mL concentration of drugs exhibited a significant cytotoxic effect during all treatment periods (Figure 2) and was selected for further assays [28,29,30].

### 2.3. Effect of Olea europaea L. Extract on Drug Efficacy

**Figure 3:**
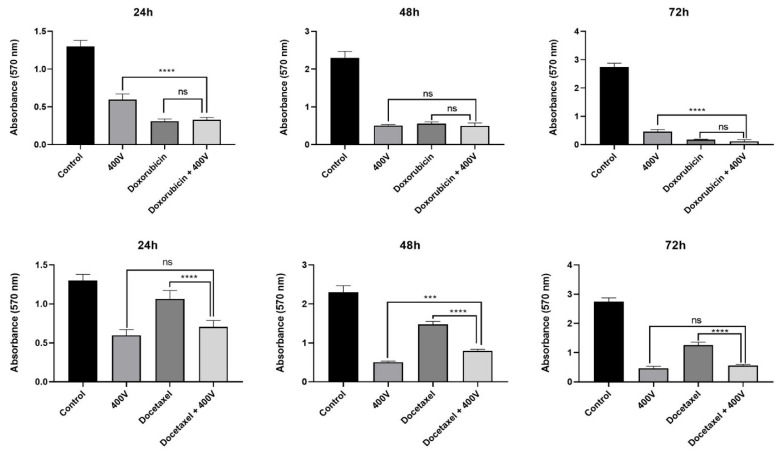

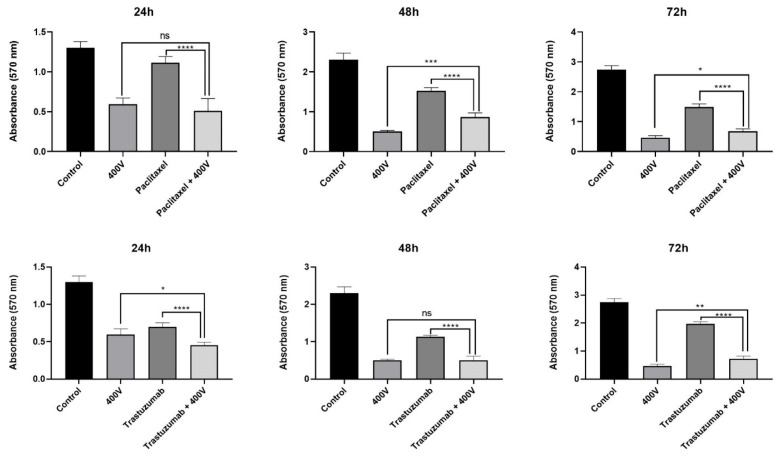
Effects of the combinations of drugs with 400 V *Olea europaea* L. extract on MCF-7 cell viability after treatment periods of 24, 48, and 72 h (ns: nonsignificant, * *p* < 0.05, ** *p* < 0.01, *** *p* < 0.001, **** *p* < 0.0001.

The potential effects of *Olea europaea* L. extract on the efficiency of drugs were also evaluated by MTT assay. According to the results, it was shown that co-treatment of MCF-7 cells with the IC_50_ dose of *Olea europaea* L. extract (400 V) and 1 µg/mL concentration of drugs significantly increased the cytotoxicity (Figure 3).

When the results were compared, doxorubicin was determined as the most effective drug, and its combination with *Olea europaea* L. extract did not significantly affect the viability of MCF-7 cells. In contrast, the viability of cells treated with only docetaxel, paclitaxel, or trastuzumab for 72 h decreased by 54.51%, 45.81%, and 28.11%, respectively. However, the addition of *Olea europaea* L. extracts to each of the docetaxel, paclitaxel, and trastuzumab treatments caused a significant decrease (*p* < 0.0001) in MCF-7 cell viability by 80.02%, 75.16%, and 73.75%, respectively.

## 3. Discussion

The inadequacy of current treatment methods has led to the search for natural and complementary factors to support cancer treatment. The role of phytotherapy because of the active compounds, particularly in the treatment of breast cancer, has recently emerged as a significant focus of research [31]. Combinations of active compounds derived from plants with cancer drugs may exhibit synergistic effects and these compounds may increase the effectiveness of the drugs [32,33,34]. Olive leaf extract has potential health benefits and positive effects on health [35,36,37]. Active polyphenolic compounds, including oleuropein, some minerals such as calcium and potassium, and vitamins such as vitamin E, which are abundantly present in olive leaves, exhibit significant antioxidant, anti-inflammatory, antimicrobial, and anticancer properties [32,38,39]. It has been reported that treatment of cells with *Olea europaea* L. extract leads to accumulation in the G0/G1 and sub-G0 phases of the cell cycle [40], as well as the induction of apoptosis associated with ROS activation in the cells [41]. Therefore, it is considered a suitable adjuvant for use in combination therapy with cancer drugs.

Upon comparing our study with the existing literature, we identified several correlations with our findings. For instance, Fares et al. [24] examined the effects of olive leaf extract (OLE) on the human lymphoblastic leukemia cell line, Jurkat. Their results indicated a 78% inhibition of Jurkat cell proliferation at a concentration of 4 μg/mL after 48 h; however, the study did not elucidate the mechanism underlying apoptosis. Furthermore, OLE has demonstrated efficacy against the myelogenous leukemia cell line K562. Following a 72 h treatment with 150 μg/mL of OLE, there was a 17% reduction in cell proliferation, accompanied by a notable decline in cell viability [22]. Similarly, an investigation into OLE’s impact on the pancreatic cancer cell line MiaPaCa-2 revealed its effectiveness in culture [42]. At a concentration of 200 μg/mL, OLE reduced cell viability to less than 1% compared to controls. Coccia et al. [43] studied the cytotoxic effects of extra virgin olive oil extract on two distinct bladder cancer cell lines, reporting a significant reduction in proliferation for both the T24 and 5637 bladder cell lines in a dose-dependent manner. For the T24 cell line, cell viability decreased by up to 90% with treatment using 100 μg/mL of the oil, indicating an IC_50_ of approximately 32 μg/mL, with similar efficacy observed in 5637 cells [43]. Additionally, cell cycle progression was assessed via flow cytometry, revealing a growth arrest at the G2/M phase following treatment in both cell lines. In the T24 cell line, there was a decrease in the G0/G1 phase and an increase in the sub-G1 fraction, suggesting the induction of apoptosis. Western blot analysis of pro-caspase-3 and -9 and PARP-1 further confirmed the apoptotic effects of the oil extract [16].

Cancer-specific calcium-induced apoptosis is a targeted approach to triggering cell death, specifically in cancer cells, while leaving healthy cells unharmed. This targeted effect is achieved by focusing on particular calcium channels or disrupted signaling pathways that are unique to cancer cells. Also, the elevated levels of extracellular potassium have been found to influence the behavior of T cells, leading them to maintain a stem-cell-like quality, also known as “stemness”, which is closely associated with their capacity to eradicate cancer during immunotherapy [44]. This suggests that increasing T cells’ exposure to potassium or replicating the effects of elevated potassium levels could potentially enhance the efficacy of cancer immunotherapies. It was shown that heightened extracellular potassium levels improved the ability to clear tumors [45].

Natural forms of vitamin E, including tocopherols and tocotrienols, serve as potent antioxidants, capable of preventing DNA damage caused by oxidative stress. Furthermore, derivatives and metabolites of vitamin E have been demonstrated to possess anti-inflammatory and potentially anticancer properties [46,47]. So, in the above consideration, the cellular mechanisms that impact calcium, potassium, and vitamin E levels could potentially be exploited for therapeutic applications.

Viscosity is also an important parameter for the transition of a substance from a solid to a liquid. The high intermolecular forces present in liquids contribute to their elevated viscosity and their effect on molecular structures [48]. These forces usually affect chemical reactions between some metabolites, minerals, and vitamins, leading to changes in the behavior of the molecules in the bonding reactions. Thus, measuring the viscosity level can provide preliminary information about the activity of natural extracts on some cells.

As mentioned above, calcium, potassium, vitamin E, and the viscosity of plant extracts are highly significant in their effects on cancer cells. This study aimed to elucidate the impact of various minerals and vitamins present in different viscous extracts, highlighting their distinct influences on breast cancer cell lines both alone and in combination with cancer treatment medications.

The analysis of minerals and vitamins reveals the remarkable properties of OWE 400 V (highest viscosity), showcasing the highest concentrations of phosphorus (218 ± 7 mg/100 g) and calcium (301 ± 12 mg/100 g) compared to the results of OWE 200 V and 300 V. Emphasizing the richness of its nutritional profile, the three different viscosities of OWE present varying levels of vitamins A, B5, C, D2, E, and K1, as illustrated in Table 1. Among the extracts, OWE 400 V has the highest vitamin E quantification with 0.36 ± 0.01 mg/100 g, 200 V has 0.08 ± 0.01 mg/100 g, and 300 V has 0.2 ± 0.01 mg/100 g (Table 1).

In this study, although *Olea europaea* L. extract did not alter the effectiveness of doxorubicin, it notably enhanced the efficacy of docetaxel, paclitaxel, and trastuzumab. An average increase of 27% in the effectiveness of docetaxel was observed across all treatment durations. In contrast, paclitaxel exhibited a notable increase of 48% in effectiveness following 24 h of treatment. Additionally, a 46% increase in effectiveness was reported for trastuzumab following 72 h of treatment. The enhancement in the effectiveness of paclitaxel and trastuzumab, which are known to have weak efficacy when used alone on cancer cells, suggests that when *Olea europaea* L. extract was combined with these drugs, the inhibition effectiveness of the cancer cells was increased by OWE, so OWE may play an essential role in guiding breast cancer treatment. Further studies involving a wider range of breast cancer cell lines to fully evaluate the potential of olive leaf extract as an adjuvant treatment.

## 4. Material and Methods

### 4.1. Extraction of Olea europaea L. Leaf

During the extraction process, natural OWE was obtained from Ilhan Sari organic olive farms in Koprubasi/Manisa, Turkey, using the small Pulveriser HL-50 and the small Extraction and Concentration Unit DT-100, ChengDong Medicine Machine, Shanghai Chengdong Technology Co., Ltd, Shanghai, China. An amount of 100 g of *Olea europaea* L. Ayvalik and Gemlik leaves was carefully gathered from the natural environment in Köprubasi/Manisa, Turkey. The collected leaves underwent natural drying away from direct sunlight and were then finely ground using a herb grinder. In the cold maceration process, 50 g of each ground sample was precisely measured and placed into the glass tube of a rotary evaporator. These glass tubes were subsequently filled with 1000 mL of water and then mixed at a gentle 5 rpm rotation speed overnight. Following this, the samples were filtered, and water was removed to reach a viscosity of 200 (200 V, lowest viscosity), 300 (300 V, medium viscosity), or 400 (400 V, highest viscosity) centipoise/s under a 5 Pa vacuum and 20 rpm rotation speed. The viscosity parameters of all studied extracts were measured using a Thermo Haake Viscotester (Thermo Fisher Scientific, Waltham, MA, USA). This unique natural extraction procedure will be patented in Turkey and other countries. The resulting OWEs were utilized as samples for the analysis of minerals (calcium (Ca), ferritin (Fe), magnesium (Mg), manganese (Mn), phosphorus (P), potassium (K), selenium (Se), sodium (Na), and zinc (Zn)) and vitamins (A, B5, C, D2, E, and K1), and for cytotoxic effect investigations on cell lines.

### 4.2. Mineral and Vitamin Analysis of OWE

The mineral (calcium (Ca), ferritin (Fe), magnesium (Mg), manganese (Mn), phosphorus (P), potassium (K), selenium (Se), sodium (Na), and zinc (Zn)) and vitamin (A, B_5_, C, D_2_, E, and K_1_) analyses in three different viscosities of OWE were conducted by procuring services from the Bursa Test and Analysis Laboratory (BUTAL). This laboratory utilizes accredited chromatographic methods approved by the Scientific and Technological Research Council of Turkey. The applied methods involved the use of HPLC for analyzing vitamins, with the exception of vitamin K1, which was analyzed using HPLC-FLD. Mineral analysis in three different viscosities of OWE (200 V, 300 V, and 400 V) were realized using ICP-OES to detect and quantify K, P, Ca, Fe, Mg, Mn, Zn, and Na. Additionally, ICP-MS was used by BUTAL to analyze selenium minerals in OWE.

### 4.3. Cell Culture

The estrogen receptor-positive (ER+) MCF-7 breast cancer cell line was obtained from the American Type Culture Collection (ATCC HTB-22; Manassas, VA, USA). The cells were cultured in high-glucose DMEM (Dulbecco’s Modified Eagle Medium, Gibco, Grand Island, NY, USA) containing 10% fetal bovine serum (FBS, Gibco, USA) and 1× penicillin–streptomycin (Gibco, USA). An atmosphere of 5% CO_2_, a temperature of 37 °C, and a relative humidity of 95% were provided for the incubation of the cells, and cell growth and viability were regularly monitored under an inverted microscope (Olympus, Tokyo, Japan). Docetaxel, doxorubicin, paclitaxel, and trastuzumab were purchased from Merck (Darmstadt, Germany).

### 4.4. MTT Assay

Cells were seeded in a 96-well plate at a density of 10,000 cells per well. An overnight incubation was conducted to enhance cell adhesion. Following this incubation, the cells were treated with various concentrations of chemotherapeutic drugs (docetaxel, doxorubicin, paclitaxel, and trastuzumab) and *Olea europaea* L. extracts with different viscosities (200 V, 300 V, and 400 V) and concentrations (50–2000 µg/mL). Cells that were untreated or treated only with medium were considered as the controls. After incubation times of 24, 48, and 72 h, 10 µL of MTT (3-(4,5-di-methylthiazole-2-yl)-2,5-diphenyltetrazolium bromide) (Sigma, St. Louis, MO, USA) solution was added to each well, and the plate was incubated for 4 h to allow the reduction of MTT. Subsequently, the formed purple formazan crystals in the wells were dissolved in 100 µL dimethyl sulfoxide (Serva, Heidelberg, Germany), and the optical density was measured at 570 nm. The obtained absorbance values were used to calculate cell viability percentages [49]. The most effective doses of chemotherapeutic drugs and *Olea europaea* L. extracts were selected for further analysis.

### 4.5. Synergistic Effects of Olea europaea L. Extracts

Cells seeded in a 96-well plate at a density of 10,000 cells per well were co-treated with the most effective selected doses of chemotherapeutic drugs and *Olea europaea* L. extracts for 24, 48, and 72 h under standard conditions. Cells in wells containing only the medium were designated as controls. Following the incubation period, the MTT assay was carried out as described before.

### 4.6. Statistical Analysis

All experiments were conducted in triplicate. Data were analyzed using GraphPad Prism v.3.0 (GraphPad Prism v.3.0, GraphPad Software, San Diego, CA, USA) using one-way analysis of variance (ANOVA) followed by Dunnett’s and Tukey’s tests. All results were presented as mean ± standard deviation and *p* < 0.05 was used to indicate a significant difference.

## 5. Conclusions

This research study thoroughly examined the effects of three distinct viscosities of OWE, mentioned as 200 V, 300 V, and 400 V, on the breast cancer cell line. This investigation focused on how these different viscosities influence the behavior of breast cancer cell lines. In addition to assessing the individual effects of the OWE, this research also evaluated the combined impact of these extracts with several widely utilized chemotherapeutic agents, namely docetaxel, doxorubicin, paclitaxel, and trastuzumab. This multifaceted approach aimed to determine whether incorporating varying viscosities of OWE could enhance the therapeutic efficacy and potential treatment outcomes for breast cancer, providing pre-information on innovative in vivo experiments for cancer therapy. The results indicated that the viscous extract of *Olea europaea* L. leaves, identified as OWE 400 V, is particularly rich in calcium, potassium, phosphorus, and vitamin E compared with the results of the OWE 200 V and 300 V. When combined with the medications docetaxel, paclitaxel, and trastuzumab, OWE 400 V enhanced the efficacy of these treatments against breast cancer cells due to its valuable minerals, vitamins, and metabolites, as well as their synergistic effects.

The results obtained provided valuable information about the effects of OWE on breast cancer. These findings show the potential of OWE in breast cancer cells and provide critical insights into the mechanisms of action, selectivity, and safety of OWE compounds. However, in-depth in vitro and in vivo experiments are essential to thoroughly investigate the inhibition mechanism of OWE in breast cancer. These experiments will help uncover the intricate details of the entire mechanism and identify the active parameters involved in this process.

## Figures and Tables

**Table 1:** Results of minerals and vitamins in OWE.

Experiments	Unit	Experimental Method	Results with ± SD		
			200 V	300 V	400 V
**Minerals**					
Potassium (K)	mg/100 g	ICP-OES	1536 ± 56	1507 ± 9	1744 ± 33
Phosphorus (P)	mg/100 g	ICP-OES	188 ± 5	202 ± 2	218 ± 7
Calcium (Ca)	mg/100 g	ICP-OES	284 ± 3	296 ± 4	301 ± 12
Ferritin (Fe)	mg/100 g	ICP-OES	5.50 ± 0.50	5.28 ± 0.21	3.74 ± 0.08
Magnesium (Mg)	mg/100 g	ICP-OES	120 ± 4	118 ± 1	118 ± 5
Manganese (Mn)	mg/100 g	ICP-OES	1.29 ± 0.03	1.30 ± 0.02	1.30 ± 0.01
Zinc (Zn)	mg/100 g	ICP-OES	16.3 ± 2.8	20.5 ± 2.0	19.8 ± 1.2
Sodium (Na)	mg/100 g	ICP-OES	85.4 ± 6.3	86.7 ± 2.4	45.8 ± 2.4
Selenium (Se)	µg/100 g	ICP-MS	6.78 ± 0.08	6.67 ± 0.09	6.87 ± 0.19
**Vitamins**					
A	mg/100 g	HPLC	<0.01	<0.01	<0.01
B_5_	mg/100 g	HPLC	< 0.3	<0.3	<0.3
C	mg/100 g	HPLC	16.2 ± 0.5	17.8 ± 0.1	13.9 ± 0.4
D_2_	mg/100 g	HPLC	<0.04	<0.04	<0.04
E	mg/100 g	HPLC	0.20 ± 0.01	0.08 ± 0.01	0.36 ± 0.01
K_1_	mg/100 g	HPLC-FLD	0.010 ± 0.001	0.010 ± 0.001	0.010 ± 0.001

SD: Standard deviation.

## Data Availability

All data are contained within the article. Any further information can be provided by the authors upon request.
